# Risk factors for survival after lung transplantation in cystic fibrosis: impact of colonization with multidrug-resistant strains of *Pseudomonas aeruginosa*

**DOI:** 10.1007/s15010-025-02478-z

**Published:** 2025-01-30

**Authors:** Bettina Weingard, Sören L. Becker, Sophie Schneitler, Franziska C. Trudzinski, Robert Bals, Heinrike Wilkens, Frank Langer

**Affiliations:** 1 Internal Medicine V, Saarland University, 66421 Homburg/Saar, Germany; 2 Institute of Medical Microbiology and Hygiene, Saarland University, 66421 Homburg/Saar, Germany; 3 Department of Thoracic Surgery, Saarland University, 66421 Homburg/Saar, Germany; 4 Thoraxklinik at Heidelberg, University Hospital, 69126 Heidelberg, Germany

**Keywords:** Cystic fibrosis, *Pseudomonas aeruginosa*, MDR, Lung transplantation, Organ allocation, Survival

## Abstract

**Background:**

Lung transplantation is the ultimate treatment option for patients with advanced cystic fibrosis. Chronic colonization of these recipients with multidrug-resistant (MDR) pathogens may constitute a risk factor for an adverse outcome. We sought to analyze whether colonization with MDR pathogens, as outlined in the German classification of multiresistant Gram-negative bacteria (MRGN), was associated with the success of lung transplantation.

**Methods:**

We performed a monocentric retrospective analysis of 361 lung transplantations performed in Homburg, Germany, between 1995 and 2020. All recipients with a main diagnosis of cystic fibrosis (*n* = 69) were stratified into two groups based on colonization with *Pseudomonas aeruginosa* in view of MRGN before transplantation: no colonization and colonization without (*n* = 23) *or* with (*n* = 46) resistance to three or four antibiotic groups (3MRGN/4MRGN). Multivariable analyses were performed including various clinical parameters (preoperative data, postoperative data).

**Results:**

CF patients colonized with multidrug-resistant pathogens (*Pseudomonas aeruginosa*) classified as 3MRGN/4MRGN had poorer survival (median survival 16 years (without MRGN) *versus* 8 years (with MRGN), *P* = 0.048). Extracorporeal support (*P* = 0.014, HR = 2.929), re-transplantation (*P* = 0.023, HR = 2.303), female sex (*P* = 0.019, HR = 2.244) and 3MRGN/4MRGN (*P* = 0.036, HR = 2.376) were predictors of poor outcomes in the multivariate analysis. Co-colonization with the mold *Aspergillus fumigatus* was further associated with mortality risk in the 3MRGN/4MRGN group (*P* = 0.037, HR = 2.150).

**Conclusion:**

Patients with cystic fibrosis and MDR colonization (*Pseudomonas aeruginosa*) are risk candidates for lung transplantation, targeted diagnostics and tailored anti-infective strategies are essential for survival after surgery. MDR colonization as expressed by MRGN may help to identify patients at increased risk to improve the organ allocation process.

## Introduction

Approximately 8.000 individuals are currently affected by cystic fibrosis (CF) in Germany, while at least 100,000 people worldwide live with this condition [31]. The median age at death is 37 years; however, the median projected life expectancy for a newborn child (born between 2019 and 2023) is 61 years [Cystic Fibrosis Foundation Registry, 2023] due to the advent of new medical strategies. Since 2020, a novel triple combination therapy (ivacaftor, tezacaftor and elexacaftor) is available for patients with F508del-mutation in CF, but lung transplantation will continue to be the final treatment option for patients with end-stage cystic fibrosis. In 1988, the Toronto Lung Transplant Program performed the first bilateral lung transplant in a patient with end-stage CF [36]. Several organ systems (lung, pancreas, liver) are affected by this disease, but pulmonary manifestations predict morbidity and mortality. Tenacious secretions that decrease mucociliary function lead to chronic lower airway infection. Accordingly, CF patients are prone to develop infections with bacterial, fungal and viral pathogens [32, 34].

The Gram-negative bacterium *P. aeruginosa* is the most commonly identified pathogen in persons with CF and is associated with morbidity and increased risk of mortality [5, 32]. Specific epidemic strains of *P. aeruginosa* can create adaptive mechanisms that cause chronic infections, such as increased protective biofilm formation, antibiotic resistance, and downregulation of acute virulence factors [14, 18, 23]. Despite advances in microbiological diagnosis and anti-infective therapy, CF treatment continues to be a clinical challenge, particularly in increasingly aging patients. Besides *P. aeruginosa*, other multidrug-resistant (MDR) Gram-negative pathogens such as *Escherichia coli* and *Klebsiella pneumoniae* are frequent causes of infections necessitating antibiotic treatment in CF patients.

The allocation of donor lungs is based on the lung allocation score (LAS) within the UNOS systems, Germany and the Netherlands. This mathematical model predicts urgency and success of lung transplantation. The pre-transplant colonization with MDR bacteria reduces antibiotic treatment options to treat pulmonary post-transplant infections. Infections are the primary causes of death within one year after lung transplantation [ISHLT database, 2019] and are associated with sepsis as most crucial factor for ICU readmission [16]. However, colonization with MDR pathogens or the necessity of therapy with newer reserve antibiotics such as ceftolozane-tazobactam or ceftazidime-avibactam is not considered in the LAS calculation.

The current monocentric analysis seeks to characterize the outcome after lung transplantation in CF patients with pre-transplant MDR colonization as compared to CF patients without MDR colonization over a 25-year period.

## Methods

### Ethics approval

The study was approved by the ethics committee of the Medical Council of Saarland, identification number: 162/19.

### Study design and data collection

Between October 1995 and August 2020, a total of 361 LUTX due to different underlying lung diseases were performed in our center. All patients with CF (*n* = 69) were included in this retrospective study, which focuses on the impact of the colonization with therapy-resistant pathogens on the survival rate after LUTX in patients with CF. The study was conducted in compliance with the Declaration of Helsinki. The CF patients were divided into two groups regarding colonization with *P. aeruginosa*: One group with colonization and *with* antibiotic resistance (*P. aeruginosa, 3MRGN or 4MRGN*) (*n* = 46). The second group with colonization but *without* antibiotic resistance (*P. aeruginosa*) or *without* colonization with *P. aeruginosa* (*n* = 23).

### Microbiological classification

To define MDR status, we adhered to the classification currently being used in Germany, which was put forth by the Committee for Hospital Hygiene and Infection Prevention at the German Robert Koch Institute (KRINKO 2012). In brief, it considers the four most commonly used antibiotic classes and considers a Gram-negative rod as “multiresistant Gram-negative” (MRGN) whenever it is resistant to either three (3MRGN) or all four (4MRGN) of these antibiotic classes. For *P. aeruginosa*, details are given in Table 1.

**Table 1:** Classification of multiresistant Gram-negative rod-shaped bacteria (*P. aeruginosa*) (KRINKO 2012). **R** = resistant or intermediate sensitivity, **S** = sensitivity

Group of antibiotics	Lead substance	*Pseudomonas aeruginosa*	
		3MRGN^1^	4MRGN^2^
Acylureidopenicillines	Piperacillin	Only one of the 4 groups of antibiotics effective	R
3rd /4th generation cephalosporines	Ceftazidime and cefepime		R
Carbapenems	Imipenem and meropenem		R
Fluoroquinolones	Ciprofloxacin		R

^1^ 3MRGN (multiresistant Gram-negative rod-shaped bacteria with resistance to 3 of the 4 groups of antibiotics)

^2^ 4MRGN (multiresistant Gram-negative rod-shaped bacteria with resistance to 4 of the 4 groups of antibiotics)

### Strategy of antibiotic prophylaxis and treatment after LUTX

For the prevention and treatment of multidrug-resistant bacterial infections, particularly *P. aeruginosa*, inhaled colistin is administered in all cystic fibrosis (CF) patients following lung transplantation in our center as a component of the post-transplant antibiotic strategy. The primary goal is to prevent the colonization of the new lung graft by MDR-pathogens [6, 7].

Sinus disease prophylaxis includes regular nasal saline irrigation. In patients with chronic sinus infections, long-term local antibiotics are administered in addition to inhaled colistin. Regular ENT (ear, nose, and throat) assessments are recommended to detect early signs of sinus involvement and to adjust treatment accordingly. Sinus surgery is performed in refractory cases to further optimize sinus drainage and prevent complications.

For detection of colonization and treatment of infections our strategy includes.

1. Post-Transplant Surveillance with regular monitoring of respiratory secretions to detect early colonization or infection according to recommendations in CF-patients [29].
2. Targeted Antibiotic Therapy: Once a colonization or infection is identified, targeted antibiotic therapy is initiated with the goal of eradication therapy with a multidisciplinary approach, including an infectious disease specialist, pulmonologist, and transplant surgeon. Tailored treatment plans are based on the patient’s specific bacterial flora, susceptibility profiles and clinical status. In patients with *P. aeruginosa* a combination of two antipseudomonal antibiotics is used.

### Clinical data

The following clinical data of the recipients were collected (see Table 2): age, sex, body mass index (BMI [kg/m^2^]), oxygen required at rest (l/min) or FiO_2_ (%) determined by oxygen titration to achieve a partial pressure of oxygen (paO_2_) = 60 mmHg, FEV_1_ (% predicted, FVC (% predicted), CRP (mg/l), ECMO or ECCO_2_R, mechanical ventilation, non-invasive or invasive ventilation, functional status (Barthel-Index), Rockwood scale for evaluation of physical, cognitive and psychological status, lung allocation score (LAS), before the implementation of the LAS in December 2011, the former High Urgency status (HU) was equated with a LAS ≥ 75), time on the waiting list, number of hospitalizations before LUTX, incidence of chronic lung allograft dysfunction (CLAD), CLAD-free survival (months), date of death, and cause of death.

**Table 2:** Characteristics of LUTX in patients with CF. Categorial characteristic are specified as number in case (%) and quantitative characteristics as median (with interquartil range)

Characteristic	CF *n* = 69	without 3MRGN/4MRGN (*P. aeruginosa*) *group 1*: *n* = 23	with 3MRGN/4MRGN (*P. aeruginosa*) *group 2*: *n* = 46	*P*
male	36/69 (52.17)	11/23 (47.82)	25/46 (54.35)	0.621
female	33/69 (47.83)	12/23 (52.17)	21/46 (45.65)	0.621
age (years)	27.00 ± 12.00	24.00 ± 14.00	27.50 ± 12.00	0.504
BMI (kg/m^2^)	17.00 ± 3.50	17.00 ± 3.50	17.05 ± 3.60	0.992
FEV_1_(%)	19.00 ± 7.60	18.10 ± 6.50	19.65 ± 8.45	0.218
Invasive ventilation	28/69 (40.58)	3/23 (13.04)	25/46 (54.35)	0.001
ECMO/ ECCO_2_R	24/69 (34.78)	2/23 (8.70)	22/46 (47.82)	0.001
LAS ≥ 75 or HU	39/69 (56.52)	8/23 (34.78)	31/46 (67.39)	0.019
waiting time (LUTX) (months)	12.30 ± 25.60	17.73 ± 20.67	6.62 ± 27.55	0.173
re-TX	8/69 (11.59)	3/23 (13.04)	5/46 (10.87)	0.402
re-re-TX	1/69 (1.45)	1/23 (4.35)	0 (0.00)	0.402
Aspergillus fumigatus	32/69 (46.37)	8/23 (34.78)	24/46 (52.17)	0.207
Aspergillus spp.	18/69 (26.09)	2/23 (8.70)	16/46 (34.78)	0.022
CLAD-free survival (months)	40.93 ± 86.47	60.37 ± 138.07	24.10 ± 74.47	0.005

BMI: Body-Mass-Index; CLAD: chronic lung allograft dysfunction, ECMO: extracorporal membrane oxygenation, ECCO_2_R: extracorporeal CO_2_ removal, FEV_1_: forced expiratory volume per second, HU: high urgency, LAS: lung allocation score, re-TX: retransplantation, re-re-TX: re-retransplantation, spp.: subspecies

### Statistical analysis

All analyses were performed with IBM^®^ SPPS Statistics 26 Software. Normal distribution was assessed using the Kolmogorov-Smirnov-test. Normally distributed data were expressed as mean ± standard deviation, non-normally distributed as median ± interquartile range. The statistical analysis included comparisons between groups (normally distributed data: t-test, non-normally distributed data: Mann-Whitney-U-test, discrete data: Fisher’s exact test. The Kaplan-Meier analyses were calculated with log-rank-test, the univariable, bivariable and multivariate Cox regressions with a confidence interval of 95% for the Hazard Ratio.

First, a univariable regression analysis was carried out with regard to MRGN and the HR was determined. Potential confounders, which caused a difference of ≥ 10% of the HR value in bivariable analyses compared to the univariable analysis, were included in multivariable analyses as covariates. No more than 4 predictors were included in the respective multivariate analysis to achieve precise statistical results (see Tables 3, 4 and 5).

**Table 3:** Multivariate survival time analysis (with predictors before LUTX) with Cox-Regression at CI 95%

Predictor before LUTX	HR	95% confidence interval for HR		*P*
		lower	upper	
*P. aeruginosa* **with 3MRGN/4MRGN**	2.376	1.058	5.335	**0.036**
Sex of recipient (female)	2.244	1.144	4.403	**0.019**
Re-/ Retransplantation	2.303	1.125	4.714	**0.023**
LAS ≥ 75 or HU	1.639	0.788	3.406	0.186

LAS: lung allocation score, HU: high urgency, HR: hazard ratio

## Results

All 69 CF patients (52% male, mean age 27 years, BMI 17 kg/m^2^) underwent bilateral LUTX. Included are 8 retransplantations (re-LUTX) and one re-re-LUTX. The mean waiting time for an organ was 1.5 ± 1.7 years. Antibiotic testing panels detected 46 of these patients being colonized with 3MRGN/4MRGN *P. aeruginosa* and 23 without MRGN.

Colonizations occurred in more than 10% of total cases were *Achrombacter xylosoxidans, Aspergillus fumigatus, Burkholderia cepacia* complex (*BCC), Candida albicans, Candida spp., Staphylococcus aureus MRSA, Stenotrophomonas maltophilia, Streptococcus spp. Aspergillus spp. other than Aspergillus fumigatus* showed a clear group difference, with a higher rate of colonisation in patients with **3MRGN/4MRGN***P. aeruginosa* (*p* = 0.022).

Severe underweight (BMI ≤ 16 kg/m^2^) was not a risk factor in the survival analysis (*P* = 0.521).

**Fig. 1:**
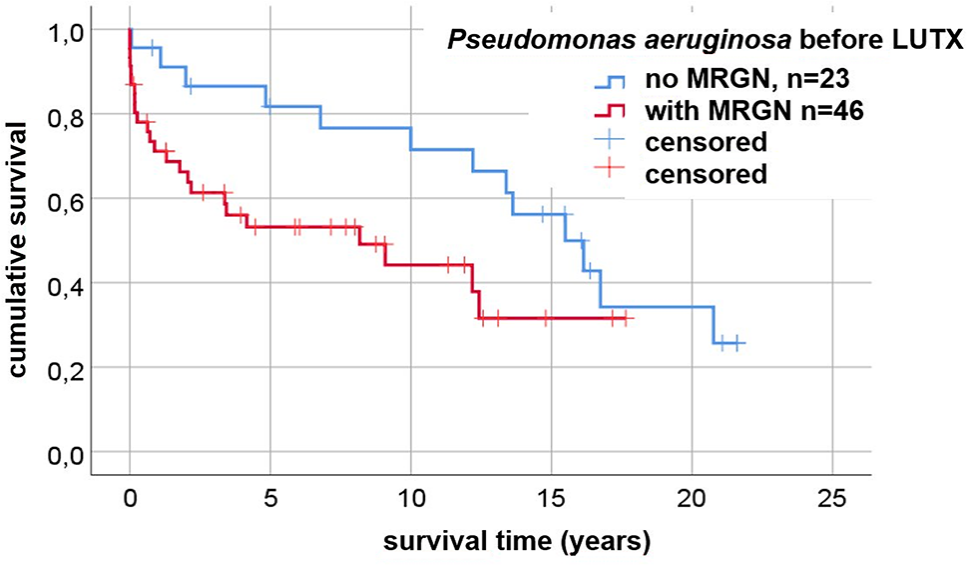
Cumulative survival of LUTX-cases in CF-patients. Log-Rank-Test, *P* = 0.048. Univariable Cox regression (MRGN): Hazard Ratio (HR) = 2.070 at CI 95% for HR: [0.994;4.308], *P* = 0.052

Patients with 3MRGN/4MRGN colonization had a poorer survival than patients without 3MRGN/4MRGN. The median survival of the whole CF-cohort was 12.16 years. Median survival in the subgroups was 8.2 years (with MRGN) versus 16.1 years (without MRGN). Survival in patients without 3MRGN/4MRGN was 96% at 1 year, 78% at 5 years and 61% at 10 years, while survival in patients with 3MRGN/4MRGN was only 72% at 1 year, 52% at 5 years and 33% at 10 years (*p* = 0.048), see Fig. 1. The CLAD-free survival was poorer in the 3MRGN/4MRGN group (24.1 months versus 60.4 months; *p* = 0.005) (See Fig. 1).

Within the follow-up 37 of the 69 patient cases died. The main cause of death was sepsis due of infection (24/37; 64.9%). Most infections were of bacterial origin without a viral or fungal component. In these 24 cases with fatal sepsis, 8 infections were based on *P. aeruginosa* with 3MRGN/4MRGN and 2 infections were due to BCC.

Other cause of death were CLAD (*n* = 4), malignancy (*n* = 2), cerebral vascular accident (*n* = 2), chronic renal failure (*n* = 2), pulmonary embolism (*n* = 1), non-occlusive mesenteric ischemia (NOD *n* = 1), surgical complication (*n* = 1).

## Multivariate survival time analysis

The negative predictors in the multivariate survival time analysis were colonization with MDR *P. aeruginosa* (3MRGN/4MRGN) before LUTX (*P* = 0.036), re-LUTX (*P* = 0.023), female sex (*P* = 0.019) and co-colonization with *Aspergillus fumigatus* (*P* = 0.037). The need for ECMO/ECCO_2_R support was the most significant life-limiting factor (*P* = 0.018, HR = 2.75) (Tables 3, 4 and 5). Colonization with *BCC* (*n* = 7: *n* = 2 (in group without *P. aeruginosa* 3MRGN/4MRGN) and *n* = 5 (in group with *P. aeruginosa* 3MRGN/4MRGN) could not detected as an risk factor, especially because of low number of cases.

**Table 4:** Multivariate survival time analysis (with predictors before LUTX) with Cox-Regression at CI 95%

Predictor before LUTX	HR	95% confidence interval for HR		*P*
		lower	upper	
*P. aeruginosa* **with 3MRGN/4MRGN**	1.787	0.739	4.319	0.197
Sex of recipient (female)	1.797	0.899	3.590	0.097
Re-/ Retransplantation	2.047	0.999	4.192	**0.050**
ECMO/ECCO_2_R	2.929	1.246	6.886	**0.014**

ECMO: extracorporeal membrane oxygenation, ECCO_2_R: extracorporeal CO_2_-removal, HR: hazard ratio

**Table 5:** Multivariate survival time analysis (with predictors before LUTX) with Cox-Regression at CI 95%

Predictor before LUTX	HR	95% confidence interval for HR		*P*
		lower	upper	
*P. aeruginosa* **with 3MRGN/4MRGN**	1.473	0.606	3.581	0.393
sex of recipient (female)	1.982	0.985	3.991	0.055
*Aspergillus fumigatus*	2.150	1.047	4.416	**0.037**
ECMO/ECCO_2_R	2.749	1.186	6.372	**0.018**

ECMO: extracorporeal membrane oxygenation, ECCO_2_R: extracorporeal CO_2_-removal, HR: hazard ratio

## Discussion

The number of patients undergoing lung transplantation worldwide has steadily increased since the end of the 1980s. According to the International Society for Heart and Lung Transplantation (ISHLT) database (2019), patients with CF have better survival than patients with other underlying lung diseases (5 year-survival 64.58% versus 60.7%; 10 year-survival 49.7% versus 42.1%). The median survival patients with CF after LUTX was 9.9 years, and the median survival in our study was 12.16 years. The better prognosis after LUTX in CF patients may be explained by the young recipient age and less relevant comorbidities (e.g. coronary heart disease). Nevertheless, CF is an insidious disease that leaves an ongoing challenge for multidisciplinary physicians involved in the care of CF patients. In the current investigation, we analyzed risk factors for survival in recipients with CF.

The prevalence of *P. aeruginosa* infection in CF increases as individual age, and early non-*Pseudomonas* airway colonizers are often supplanted by *P. aeruginosa*; until 80% of the adult patients in CF develop a chronic *P. aeruginosa* infection and generally persists indefinitely [23]. The greatest colonization of *P. aeruginosa* occurs worldwide. However, there is limited information available on the antibiotic resistance of these Gram-negative bacterial strains. In the current investigation we documented the impact of colonization with MDR *P. aeruginosa* as being associated with mortality rates after LUTX. Co-colonization with *Aspergillus fumigatus* appeared to be a negative predictor for survival after LUTX in patients with CF. Previous studies have shown that the combined colonization with *P. aeruginosa* and *Aspergillus fumigatus* is associated with a poor prognosis [20, 25, 26]. Particularly colonization with *A. fumigatus* may carry a significant risk to develop invasive aspergillosis [20, 25, 26]. Several studies showed that the infection with BCC is associated with poor prognosis and high mortality risk after LUTX. The Toronto Lung Transplant Program documented a striking difference in survival between BCC-positive and BCC-negative CF patients after LUTX (Survival in BCC-negative: 94% at 1 year, 70% at 5 years and 53% at 10 years; BCC-positive: 59% at 1 year, 33% at 5 years and 16% at 10 years; *P* < 0.001) [36]. Accordingly, many transplant centers do not accept recipients colonized by BCC [8–10, 13, 21]. Surprisingly, colonization with BCC could not be detected as a risk factor for death in our cohort. One recipient, who is currently 62 years old, is alive 26 years post-transplant and was colonized with BCC prior to lung transplantation.

The ISHLT database (2019) demonstrates that female sex is associated with superior survival after LUTX. In CF-patients, however, survival after LUTX is better in male recipients. The sex dimorphism may be explained by female hormones, the effect is characterized as “CF gender gap” [11, 12, 35]. Estrogenes are capable of modifying epithelial sodium channels, which leads to a reduction of the airway surface liquid and worsening of mucociliary clearance. Therefore, the chloride channel protein CFTR is not the only clinical complication in females. In addition, estrogens enhance the virulence of *P. aeruginosa*, this may contribute to lung exacerbations in women with CF [35]. Microbiological colonization or infections are associated with a decline in lung function and may aggravate sex differences. These considerations could also explain the sex effect in this study [33, 35].

Most patients with CF are cachectic due to the associated maldigestion. Malnutrition is considered as risk factor for outcome after LUTX [13]. Another study shows that patients with underweight (BMI < 17 kg/m^2^) did not have a higher mortality risk. Underweight patients with CF have acceptable survival following lung transplantation [24]. Our current analysis, however, did also not identify body mass index (BMI) as risk factor in the survival analysis, even the group with severe underweight (BMI ≤ 16 kg/m^2^) in comparison to the group with BMI > 16 kg/m^2^ (*P* = 0.521).

Re-LUTX are associated with high risk and have a poorer prognosis [4]. According to the ISHLT database, the median survival in double lung transplantation for different lung diseases is 7.8 years. The Toronto Lung Transplant Program reported a survival of 73% at 1 year, 47% at 5 years and 30% at 10 years for retranstransplant patients [36]. Our data confirm these results - retransplantation was identified as risk factor in our multivariate Cox-regression.

Extracorporeal lung support is occasionally required as bridge to LUTX. ECMO-associated risks include sepsis, bleeding and thrombosis. LUTX from ECMO is associated with a higher mortality, but may be safely performed in experienced centers (Toronto: 1 year survival 81%, 5 year survival 49%) [36]. In our previously published series survival was similar in ECMO-supported recipients and in standard LUTX-recipients [15]. In our current study, pretransplant ECMO-therapy was the most life-limiting factor and it exceeded the significance of all other covariates (colonization with *P. aeruginosa*, sex and retransplantation) in the multivariable survival time analysis.

The waiting time for LUTX depends on the LAS. The purpose of the LAS is the representation of the clinical status and the likelihood of a successful lung transplantation. We observed a significant LAS difference between the patient groups with or without 3MRGN/4MRGN with higher LAS in 3MRGN/4MRGN colonized recipients (*P* = 0.019). Although intravenous antibiotic treatment must be provided in the LAS request data panel, it is not included in the LAS calculation.

Most treatment recommendations against *P. aeruginosa* are based on expert opinion, resulting in considerable variation in antibiotic-prescribing practices among transplant clinicians. As the resistance to antimicrobial therapy increases, resistance in Gram-negative pathogens creates a serious therapeutic challenge.

Ceftolozane-tazobactam (approved since 2015 in Germany) and ceftazidime-avibactam (approved since 2017 in Germany) are applicable antimicrobials against resistant *P. aeruginosa* and can both be used effectively against multiresistant *P. aeruginosa* [1]. As most patients presented in our cohort did not receive any of these two drugs, no time trends towards an e.g. better outcome could be observed. However, there is a need for prospective surveillance in the future. Continuously improved national evidence-based guidelines are developed to improve possible treatments and outcomes in infection with multidrug-resistant *P. aeruginosa* [29].

*P. aeruginosa* biofilms are complex structures that become even more intricate when they are formed together with other microorganisms. A continuous development and improvement of efficient antibiofilm strategies are necessary to tackle the recurrence and chronicity caused by *P. aeruginosa* biofilm infections [3]. Continued high resolution surveillance, integrating phenotypic and genomic data, is required for comprehension resistance trends [28].

The microbiological mechanism leading to the conservation of a diverse microbiome in late-stage disease are still unclear as well as the impact of the function of the associated single microbes. Further research is essential to understand the overall microbiome or resistome in the response to therapy [2].

### Limitations

The present retrospective investigation represents a single-center experience of a low-volume transplant center (approximately 20 LUTX/year). Even though the observation period of the study was about 25 years the number of included patients with CF was limited. Moreover, we did not include donor characteristics in the current investigation. Additionally, our study design was retrospective, and hence, no causal relation can be inferred.

Another limitation of our study is that our data do not permit an investigation into the impact of antibiotic therapy on patient outcomes. The availability and types of antibiotic substances have changed significantly over the past 25 years. Owing to the limited number of patients, the influence of individual antibiotic groups, particularly the impact of newer antibiotic substances, could not be assessed. This should be addressed in a prospective multicenter study. Our findings of a clear difference with regard to survival between MDR-colonized and MDR-free patients should be further investigated in prospective clinical studies with a focus on development of treatment strategies.

## Conclusions

We conclude that a comprehensive consideration of the preoperative covariates in CF-patients may have impact on clinical outcome after LUTX. ECMO and re-transplantation were the most important factors in our series. If colonization with 4MRGN is documented in a recipient, intensified communication between surgeons, intensive care physicians, clinical microbiologists and infectious disease specialists should be made to develop tailored, individualised treatment strategies for a bail-out after LUTX. Considering the 3MRGN/4MRGN status associated with intensive antibiotic treatment could be helpful in better characterizing the precise clinical condition of CF recipients, which could aid in optimizing organ allocation.

## Data Availability

No datasets were generated or analysed during the current study.

## Abbreviations

A.: *Aspergillus*
BCC: *Burkholderia cepacia* complex
CI: Confidence interval
CF: Cystic fibrosis
CLAD: Chronic lung allograft dysfunction
CRP: C-reactive protein
ECMO: Extracorporeal membrane oxygenation
ECCO_2_R: Extracorporeal CO_2_ removal
FEV_1_: Forced expiratory volume per second
FiO_2_: Fraction of inspired oxygen
FVC: Forced vital capacity
HR: Hazard rate
HU: High urgency
ISHLT: International society for heart and lung transplantation
LAS: Lung allocation score
LUTX: Lung transplantation
MDR: Multiple drug resistance
MRGN: Multiresistant Gram-negative bacteria
NOD: Non-occlusive mesenteric ischemia
*P.*: *Pseudomonas*
re-LUTX: Retransplantation (Lunge)
re-re-LUTX: Re-Retransplantation (Lunge)
spp.: Subspecies
UNOS: United Network for Organ Sharing
